# Status on stroke and stroke care in Europe 2023: Stroke Service Tracker 2023 data based on 1,460,360 strokes in 47 European nations

**DOI:** 10.1093/esj/aakag008

**Published:** 2026-02-19

**Authors:** Hanne Christensen, Francesca Romana Pezzella, Melinda Berg Roaldsen, Ales Tomek, Christian Ovesen, Arlene Wilkie, Hrvoje Budincevic, Martin Dichgans, Urs Fischer, Josefine Grundtvig, Peter Kelly, Grethe Lunde, Chris Macey, Robert Mikulik, Amira Rosenørn, Gustavo Santo, Diana Aguiar de Sousa, Cristina Tiu, Simona Sacco, Aleksandras Vilionskis, Bo Norrving, SAP-E collaborators, Mentor Petrela, Marine Balasanyan, Nune Yeghiazaryan, Stefan Kiechl, Wilfried Lang, Rahim Aliyev, Nara Hesen, Sergey Marchenko, Yaroslav Zholnerkevich, Tanya Pavlovskaya, Sylvie DeRaedt, François Delvoye, Laetitia Yperzeele, Renata Jurina, Nevena Mahmutbegović, Marija Bender, Dorina Dobreva, Dimitar Maslarov, Timitar Taskov, Pere Cardona, Esther Duarte, Hrvoje Budincevic, Ervin Jančić, Maša Ožegović, Marian Charalambous, Andreas Charidimou, Georgios Kaponides, Magdalena Pohnanová, Aleš Tomek, Dorte Damgaard, Birgitte Hysse Forchhammer, Troels Wienecke, Sarah Belson, Jatt Khaira, Joseph Kwan, Janika Kõrv, Riina Vibo, Pauli Ylikotila, Susanna Roine, Guillaume Turc, Charlotte Cordonnier, Jean Bouchard, Alexander Tsiskaridze, Nina Lobajanidze, Jürgen Faiss, Markus Wagner, Tobias Neumann-Haefelin, Christina Franzisket, Haralampos Milionis, Hariklia Proios, András Folyovich, Tamás Jarecsny, Marianne Klinke, Thorir Steingrímsson, Björn Thorarinsson, Rónan Collins, Ciara Breen, Rani Barnea, Natan Bornstein, Pnina Rosenzweig, Massimo Del Sette, Fabrizio Pennaccehi, Danilo Toni, Paola Santalucia, Paolo Candelaresi, Ettore Nicolini, Zauresh Akhmetzhanova, Sabina Medukhanova, Adilbekov Yerzhan, Dren Boshnjaku, Fisnik Jashari, Kunduz Karbozova, Asel Kerimkulova, Dzhamalbek Turgumbaev, Guntis Karelis, Evija Miglane, Dalius Jatuzis, Aleksandras Vilionski, Maria Mallia, Maria Bonello, Natalia Ciobanu, Stanislav Groppa, Daniela Efremov, Milovan Roganovic, Sandra Vujovic, Heleen den Hertog, Bert Vrijhoef, Anita Arsovska, Maja Bozinovska Smicheska, Gordana Dimeska, Elena Lichkova, Fiona Quigg, Malcolm Wiggam, Annette Fromm, Agnethe Eltoft, Michal Karlinski, Adam Kobayashi, Diana Aguiar de Sousa, Elsa Azevedo, Ana Catarina Fonseca, Ana Nunes, Gustavo Santo, Diana Wong Ramos, Elena Terecoasa, Cristina Tiu, Mary Joan MacLeod, Pamela Maclean, Matthew Lambert, Ivan Milojevic, Željko Živanović, Nikola Vukašinović, Zuzana Gdovinova, Vladimir Nosal, Peter Turčáni, Bojana Zvan, Maria del Mar Freijo, Elena Lopez-Cancio, Mia von Euler, Signild Åsberg, Timo Kahles, Krassen Nedeltchev, Attila Özcan Özdemir, Mehmet Topcuoglu, Yuriy Flomin, Marina Gulyayeva, Dmytro Lebedynets, Christine Owen

**Affiliations:** Department of Neurology, Copenhagen University Hospital, Bispebjerg Hospital, Copenhagen, Denmark; Neurosciences Department, Azienda Ospedaliera San Camillo Forlanini, Rome, Italy; Department of Clinical Medicine, UiT The Arctic University of Norway, Tromsø, Norway; Clinical Research Department, University Hospital of North Norway, Tromsø, Norway; Department of Neurology, Motol University Hospital, Prague, Czech Republic; Department of Neurology, Copenhagen University Hospital, Bispebjerg Hospital, Copenhagen, Denmark; Stroke Alliance for Europe, Brussels, Belgium; Stroke and Intensive Care Unit, Department of Neurology, Sveti Duh University Hospital, Zagreb, Croatia; Department of Neurology and Neurosurgery, Faculty of Medicine Osijek, Osijek, Croatia; Institute for Stroke and Dementia Research, LMU Munich, Munich, Germany; Department of Neurology, University Hospital Bern, Bern, Switzerland; Department of Neurology, Copenhagen University Hospital, Bispebjerg Hospital, Copenhagen, Denmark; Department of Neurology, Mater Misericordiae University Hospital, Dublin, Ireland; Stroke Clinical Trials Network Ireland, Catherine McAuley Centre, Dublin, Ireland; Stroke Alliance for Europe, Brussels, Belgium; Stroke Alliance for Europe, Brussels, Belgium; Department of Neurology, St. Anne’s University Hospital, Brno, Czech Republic; Department of Neurology, Copenhagen University Hospital, Bispebjerg Hospital, Copenhagen, Denmark; Department of Neurology, University Hospital of Coimbra, Unidade Local de Saúde de Coimbra, Coimbra, Portugal; Stroke Center, University Hospital Center of Central Lisbon, Lisbon, Portugal; Faculdade de Medicina, Universidade de Lisboa, Lisbon, Portugal; Department of Neurology, University Hospital Bucharest, Bucharest, Romania; Department of Clinical Neurosciences, Carol Davila University of Medicine and Pharmacy, Bucharest, Romania; Department of Biotechnological and Applied Clinical Sciences, University of L'Aquila, L'Aquila, Italy; Clinic of Neurology and Neurosurgery, Vilnius University, Vilnius, Lithuania; Department of Clinical Sciences, Lund University, Lund, Sweden

**Keywords:** epidemiology, Europe, stroke, stroke care, stroke services, stroke treatment

## Abstract

**Introduction:**

Inequity in stroke care in Europe has previously been reported despite the existence of evidence-based cost-effective interventions for both prevention and treatment. This report aims to provide comprehensive data on stroke care in Europe in 2023 and explore associations to European regions, organisational and economic factors.

**Patients and methods:**

The Stroke Service Tracker, an annually collected survey-based dataset of aggregate summary data from participating European nations, was used with key performance indicators (KPIs) from the Stroke Action Plan for Europe (SAP-E). European regions were defined based on United Nations Geoscheme regions. Gross domestic product (GDP) per capita and healthcare spending per capita were collected based on World Bank data.

**Results:**

A total of 1,460,630 stroke events were reported in 2023 from 52 nations. Significant inequity was present in all data points and data quality varied significantly. Twenty (43.5%) nations have a national stroke plan in place, and in 19 (41.3%) work is ongoing. Quality programmes have been implemented in 20 (43.5%) nations. Implementation of a national stroke plan or a quality programme was associated with achieving more SAP-E KPIs (*P* < .001). There were regional differences in the number of met KPIs (*P* < .001). Both GDP and healthcare spending per capita were strongly correlated to meeting KPIs.

**Conclusion:**

The inequity in stroke care persists in Europe. Implementation of a national stroke plan and a quality programme is associated with meeting more KPIs; however, economic factors may pose limitations. Access to high-quality data will support decision-making towards evidence-based and cost-effective care.

## Graphical Abstract

**Graphical Abstract:**
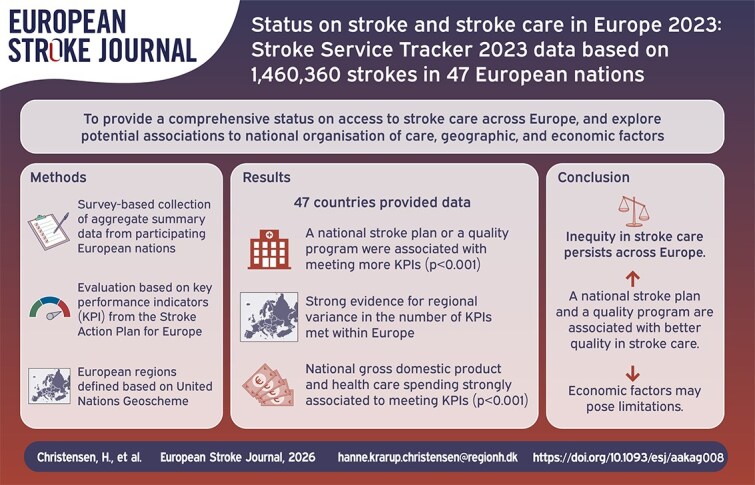

## Introduction

Stroke remains a leading cause of morbidity and mortality in Europe. Despite overall decreasing incidence rates, the number of strokes is increasing due to an ageing population.^1^ Unequal access to care in Europe has previously been demonstrated^2,3^ despite evidence-based interventions to prevent and treat stroke, which are cost-effective and recommended by WHO^4–6^ and the EU.^7^ Costs of stroke in Europe in 2017 were reported at the level of €60 billion.^8^

The aim of this report, based on 2023 data from the Stroke Service Tracker (SST) of the Stroke Action Plan for Europe (SAP-E), was to provide a comprehensive status on access to stroke care across the entire chain of care in Europe, and explore potential associations to national organisation of care, national quality programmes, healthcare spending, gross domestic product (GDP) and geographic European region.

## Methods

The data for this report were collected as part of the SAP-E 2018–2023.^9^ The SAP-E followed^10^ up on the previous Helsingborg Declaration from 1995 and 2006 and was created as a collaboration of the European Stroke Organisation (ESO) and the Stroke Alliance for Europe (SAFE). Seven domains covering the entire chain of stroke care (Primary Prevention, Organisation of Stroke Care, Quality and Outcome Assessment, Acute Care, Secondary Prevention, Rehabilitation and Life after Stroke) were included into the plan. In 2019, an implementation committee was established to set up an implementation programme to meet the SAP-E targets for 2030; this project remains the largest policy project to improve quality and access to stroke care in Europe.^11^ Fifty-two countries in WHO region of Europe have appointed National Coordinators (NCs) for SAP-E.^9^

Stroke Action Plan for Europe^9^ has developed several tools to support this implementation in individual countries including the SST.^12^ The SST is a survey-based tool designed to capture basic data on the quality of stroke care, enabling comparisons within and between countries, as well as over time. Data have been collected annually since 2020.

### Data collection

National Coordinators that are leading stroke scientific experts and representatives from stroke support organisations have been appointed by stroke scientific societies and stroke support organisations (if existent) in all countries. The SST is based on aggregate summary data; this format was chosen as it allows for exchange of data without data protection restrictions. The data represent best available data according to the NCs based on their knowledge as local experts. Data are uploaded and stored in a database hosted by the Capital Region of Denmark. Only the project leadership can access the entire data in the database. Each country has a unique access code and activities in the database are logged.

### Data items and quality

The SST includes 13 key performance indicators (KPIs) (Table S1) and basic stroke variables (eg, number of ischaemic strokes). The dataset also includes organisational survey questions (eg, an implemented national stroke plan), and the answers must be supported by documentation (uploaded documents or links). For each data point, NCs enter data source (eg, reimbursement registry).

Entries in the SST are monitored by the SAP-E organisation—each nation’s data were reviewed by two stroke experts—and queries sent back to NCs. Data are accepted as complete after return of adequate responses. All queries received response from NCs. Data sources are defined at data entry and include national/regional registries (with or without auditing and cross-checking), reimbursement registries, institution-based registries (RES-Q and SITS), direct contact to sites and estimates. Data quality was categorised as high if based on national registries including reimbursement registries, whereas individual contact to sites, estimates and extrapolation from institutional registries were categorised as lower data quality.

### Definition of variables included in this analysis

A national stroke plan (KPI 1) is reported if confirming “*A national stroke plan defining pathways, care and support after stroke, including pre-hospital phase, hospital stay, discharge and transition, and follow-up*” and providing documentation, eg, an official link. A quality programme (KPI 4) is reported if confirming “*Establishment of national- and regional-level systems for assessing and accrediting stroke clinical services, providing peer support for quality improvement, and making audit data available to the public*” and providing documentation, eg, an official link. Definitions of all KPIs are provided in Table S1.

Number of strokes (ischaemic, haemorrhagic, all strokes) are based on reported counts. A crude stroke incidence (strokes [first and recurrent] per 100,000 inhabitants) was calculated based on the reported country-specific total number of strokes in 2023 and the number of inhabitants in said country in 2023. Data on access to care (stroke unit care, intravenous thrombolysis including median door-to-needle times [DNT], mechanical thrombectomy including door-to-groin times [DGT]) were reported by NCs as total numbers (alternative as percentage if estimates) and graded according to high- or lower-quality data. Mortality within 30 days after stroke onset including mortality before discharge were reported by NCs as total numbers (alternative as percentage if estimates) and graded according to high- or lower-quality data. We pragmatically defined mortality (all stroke, ischaemic stroke and haemorrhagic stroke) within 30 days as mortality at the latest known time-point in this interval—this could include death during hospital stay if this were the only known statistic.

United Nations (UN) Geoscheme groups^13^ were used for analysing impact of region. Healthcare spending per capita (newest available)^14^ and GDP per capita^15^ were retrieved from World Bank Open Data.

### Statistical analysis

Presented data are aggregate summary data. Counts (percentage), means (standard deviation [SD]), or medians (interquartile range [IQR]) are reported as appropriate. Data for each KPI were presented using bar charts stratified according to data quality. To ensure transparency about data quality, information is presented on number of nations providing data on the specific data point, and the distribution of high-quality data or lower-quality data.

The association between categorical variables (eg, national stroke plan in place, having a quality programme as well as the geographic location in Europe) and the median number of KPI fulfilled was visualised using box plots (displaying median and lower/upper quartile) and associations were tested using the Kruskal–Wallis test.

The strength of the linear association between number of KPIs fulfilled and health expenditure per capita or GDP per capita, respectively, was quantified using the Pearson correlation coefficient. The hypothesis that the correlation coefficients were different from zero was tested by performing 10,000 permutation re-samples of the dataset. All analyses were conducted using Stata 18.0 (StataCorp, TX, USA).

## Results

In 2023, 47 countries provided data (Albania, Armenia, Austria, Azerbaijan, Belarus, Belgium, Bosnia, Bulgaria, Catalonia, Croatia, Cyprus, Czechia, Denmark, England, Estonia, Finland, France, Georgia, Germany, Greece, Hungary, Iceland, Ireland, Israel, Italy, Kosovo, Latvia, Lithuania, Malta, Montenegro, Netherlands, North Macedonia, Moldova, Northern Ireland, Norway, Poland, Portugal, Romania, Serbia, Scotland, Slovakia, Spain, Sweden, Switzerland, Turkey, Ukraine and Wales) and participated in the SST survey. Five countries did not provide data (Kyrgystan, Russian Federation, Luxembourg, Slovenia and Kazakhstan).

A total of 1,460,630 stroke events were reported in 2023: 1,016,289 ischaemic stroke events and 170,701 haemorrhagic stroke events. The crude incidence of reported strokes (Figure S1) showed regional variation; lowest incidence was reported in Albania and highest in Bulgaria.

**Figure 1:**
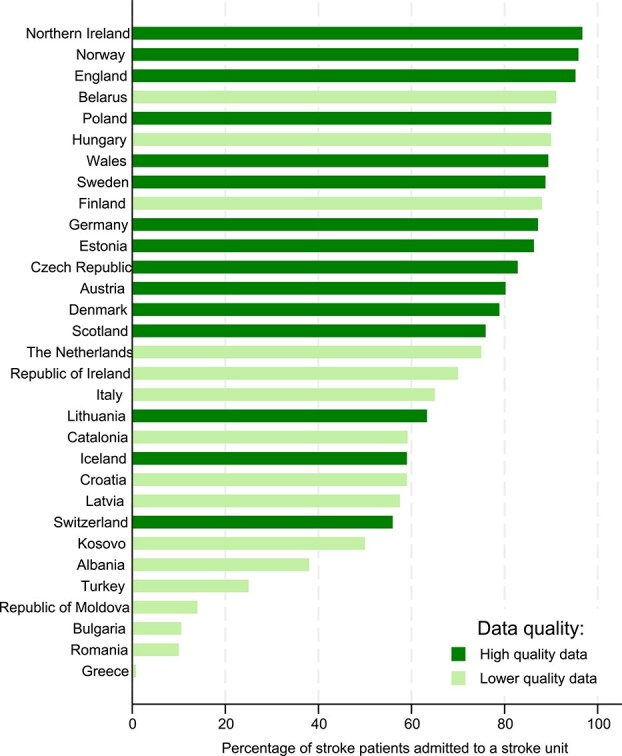
National rates of stroke unit admission.

The fraction of stroke patients admitted to stroke unit care varied significantly (Figure 1) and ranged from 0.8% to 96.7%. The European mean (SD) was 65.4% (28.2%) (31 nations provided data). Data on admission to stroke unit care within 24 h of hospital arrival were provided by 20. No country provided access to stroke unit care within 24 h of hospitalisation for at least 90% of patients. Four (20.0%) countries reported that at least 75% of patients were admitted to stroke unit care within 24 h of hospitalisation.

**Figure 2:**
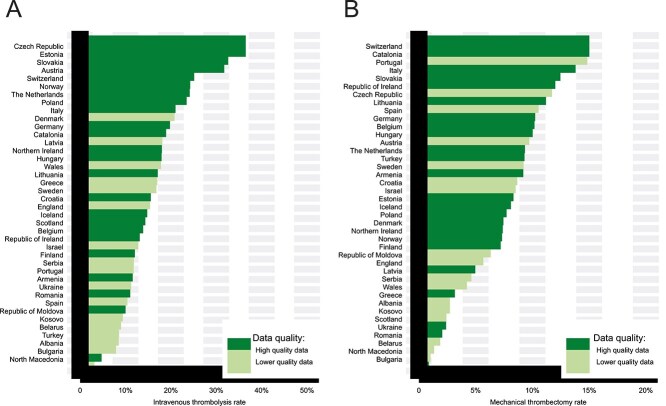
National rates of intravenous thrombolysis treatment (panel A) and mechanical thrombectomy (panel B).

Intravenous thrombolysis (IVT) rates ranged from 1.3% (North Macedonia) to 34.7% (Czechia) (Figure 2A) with a European mean (SD) of 14.3% (7.30%) (40 nations provided data). Door-to-needle times (DNT) (Figure S2a) varied highly between countries; the European median (IQR) DNT was 43 (31–59) min. Rates of mechanical thrombectomy (MT) ranged from 0.15% (Bulgaria) to 14.3% (Switzerland) (Figure 2B) with a European mean (SD) of 6.7% (3.95%) (40 nations provided data). Door-to-groin times (DGT) (Figure S2b) also varied highly between countries; the European median (IQR) DGT was 82 (64–105) min.

The European mean (SD) of all stroke mortality within 30 days was 12.9 (3.7)%—lowest in Switzerland (6.3%) and the highest in Ukraine (23.9%) (Figure S3a). In ischaemic stroke, the European mean (SD) of mortality within 30 days was 10.8 (4.0)% (Figure S3b). Mean (SD) European mortality within 30 days after haemorrhagic stroke was 31 (8.6)% (Figure S3c).

**Figure 3:**
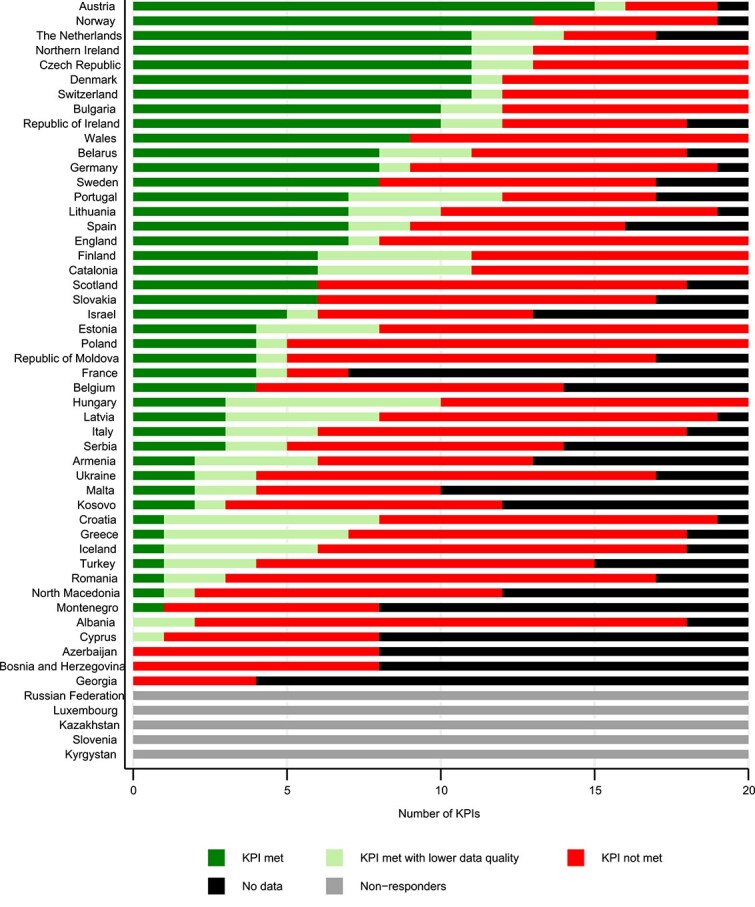
Number of KPIs met per country. In KPIs with subclassification, each response is counted as one. Abbreviation: KPIs = key performance indicators.

A national stroke plan (KPI 1) exists in 20 nations (43.5%), work is ongoing in 19 (41.3%) and work has not yet been initiated in 7 countries (15.2%). Six countries did not respond. In 20 (43.5%) nations, a quality programme (KPI 4) has been established. Six countries did not respond. The variation in meeting the SAP-E KPIs (Figure 3) is large with Northern and Central European countries generally meeting more KPIs. Meeting the individual KPIs also varies, with KPIs relating to care, rehabilitation, and swift treatment being hardest to meet (Figure S4).

**Figure 4:**
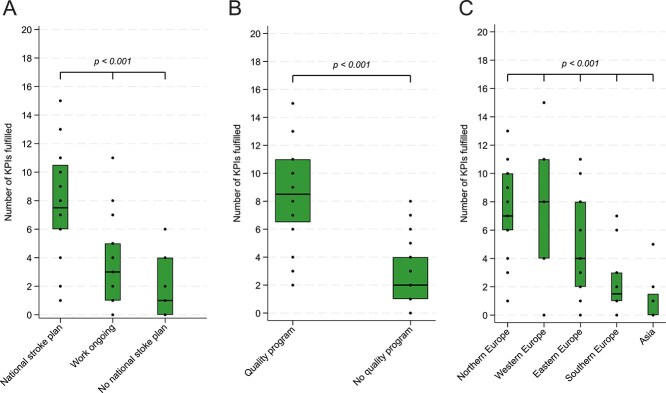
Number of KPIs met based on the implementation status of a national stroke plan (panel A), a comprehensive quality programme (panel B) or United Nations Geo-regions in Europe (panel C). Boxes represent median and interquartile range. Abbreviation: KPIs = key performance indicators.

Strong evidence was observed (*P* < .001) that having a national stroke plan in place is associated with a higher number of KPIs met (Figure 4A), in comparison to ongoing development or no national stroke plan. Having a national stroke quality programme seems to affect meeting KPIs even stronger (*P* < .001) (Figure 4B). Strong evidence was observed for geographic variation (*P* < .001) in meeting SAP-E KPIs based on European UN geoscheme regions (Figure 4C) with Northern Europe and Western Europe being at the top, followed by Eastern Europe and Southern Europe.

**Figure 5:**
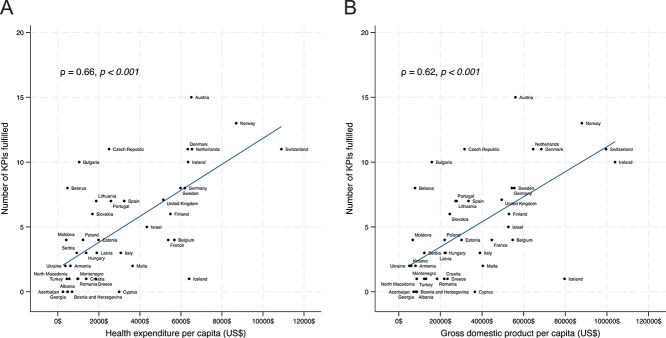
Correlation between the number of KPIs met and healthcare spending per capita (panel A) as well as gross domestic product per capita (panel B). Abbreviation: KPIs = key performance indicators.

Strong evidence for a correlation between healthcare spending per capita and meeting KPIs (ρ = 0.66, *P* < .001, Figure 5A) as well as between GDP per capita and meeting KPIs (ρ = 0.62, *P* < .001, Figure 5B), was observed.

## Discussion

We report significant variation in organisation, and inequity in access to care, and mortality across Europe in 2023. These differences are strongly associated with implementation of a national stroke plan and a stroke quality programme but also with geographic region and economic factors including GDP and healthcare expenditure per capita.

Admission to stroke unit care reduces risk of disability and death for all patients with most benefit for the severely affected and the old.^16^ The main mode of action is reducing risks of complications through mobilisation and specific interventions. This underlines the need to increase focus on access to timely stroke unit care as well as rehabilitation, as availability of stroke unit beds/care is presently insufficient in most European countries.

The overall access to acute treatments in 2023 (IVT 14.3% and MT 6.7% of all patients with ischaemic stroke) is assuring but there are massive variations between countries, and some significant variations are not easily explained only by financial factors or region. This is also relevant for treatment delays: DNT and DGT. Acute recanalisation treatments are highly time sensitive, and delays happen at the cost of neuronal survival and thereby patient outcomes and the overall benefit of treatments. These deficiencies are organisational and should be mitigated accordingly.

Mortality early after stroke varies significantly across Europe. Stroke unit care reduces mortality^17^ but the organisation and provision of care vary potentially based on conflicting stroke unit definitions as well as (lack of) resources and traditions. To reduce excess mortality in subacute stroke, it is crucial to ensure that stroke units comply with the existing evidence on organised stroke unit care.^16,18^ Organised stroke unit care is a specialised, complex, multidisciplinary intervention and organised stroke unit care requires more resources and training than a general ward. Mortality as reported by the SST may also be artificially low or high if registration by a stroke registry is reserved to stroke units and admission to stroke unit is not mandatory for all stroke patients as first level of care based on selection bias. Low mortality may result from admitting patients with no options of acute interventions to less specialised institutions, such as frail elderly patients and severely affected patients with a higher expected mortality—despite potential benefit from stroke unit care.^19^ Higher mortality may result from not admitting very mild strokes to stroke units and thereby enabling registration.

The observed relation between quality of care and implementation of a national stroke plan is not unexpected: An implemented national stroke plan ensures defined treatment pathways, a resilient organisation and funding. Also, a quality programme was shown to be strongly related to quality of care. A quality programme is an integrated part of a national stroke plan and as the quality programme promotes access to care and quality of care, the stroke plan and the quality programme are strongly correlated. In most cases, the national stroke plan will precede implementation of a quality programme which may explain the apparent larger impact of a quality programme. In 2023, national stroke plans were being implemented at high speed in European countries and significant optimism is appropriate. It should, however, be cautiously monitored if national stroke plans will ensure adequate high-quality data acquisition and quality control. Accurate data are indispensable in oversight of the treatment needs in the population, quality of care, and to support prioritisation of evidence-based and cost-effective interventions.

Economic factors—GDP and healthcare spending per capita—are also strongly correlated to quality of care. There is no doubt that adequate funding of healthcare systems is needed to ensure quality in care; however, it is interesting to observe that there is a close overlap between the countries performing best in meeting SAP-E KPIs and those that perform above their healthcare spending. This may reflect the potential health economic benefits from prioritising evidence-based interventions or simply cost-effective organisation. Such comparisons may motivate healthcare systems to learn from each other, which may ultimately benefit patients as well as societies. The geographic differences in meeting KPIs are highly significant but also covers the fact that variation is also significant within the regions. This may form the basis for sharing knowledge and experience between countries on efficient organisation of stroke care.

The strength of this analysis is its comprehensiveness and completeness—it is based on 47 nations—allowing for an overview of the status of stroke care in Europe. Data derive from local experts in each country and represent best available data. Data sources are declared in detail, allowing data quality to be included in the interpretation. The use of aggregate summary data has permitted collection of data without GDPR concerns. There are, however, some limitations. Not all countries in EU-53 have reported data (Russia, Slovenia, Kyrgyzstan, Kazakhstan and Luxembourg). Pendres et al.^1^ reported a total of 1,802,560 incident stroke in EU-53. Based on the SST dataset from 2023, we report a total of 1,460,360 strokes. Disregarding the likely declining incidence of stroke, the main reason is the lack of data from the 5 nonparticipating countries.

The use of aggregated summary data does not permit correcting for individual patient factors that may vary between countries, eg, risk factor presentation or age. We have attempted to mitigate the risks from using survey methodology, which does not allow for monitoring source data by collecting documentation, eg, for stroke registries, as well as by monitoring data and sending queries whenever relevant. The variations in data quality also remain a limitation, as data from selected institutions, as well as estimates, are included; however, data quality is clearly presented, ie, in graphics for transparency. Another significant limitation remains that this dataset is based on patients who have been documented in national registries, and only a few of them report stroke registry coverage exceeding 90%.

## Conclusion

This analysis of 2023 SST data from 47 European countries shows progress in access to acute stroke treatments, however, substantial gaps persist, largely driven by economic constraints and insufficiently developed healthcare systems. There is an obvious need in most countries for more focus on stroke unit care, rehabilitation and follow-up to reduce the burden of stroke on societies and people by improving outcomes and thereby increasing the chance for independent living after a stroke.

It is very positive that national stroke plans are being implemented at high speed in Europe, with a likely impact in the next few years. Stroke care is cost-effective, and the burden of stroke remains high in Europe. A significant limitation of the inferences drawn from the data presented in this study remains that some countries were only able to provide estimates from low-quality data sources and should hence be interpreted with caution. As high-quality data should be the basis of informed decision making towards evidence-based and cost-effective treatments and care, this gap should be closed by high-level initiatives using resilient electronic solutions with a focus on key variables along the entire chain of care.

The impact of economic factors on the provision of care is significant, as well as the regional differences. Support for specific countries, eg, as peer-to-peer support from countries with successful developments, may prove to be a useful approach.

## Supplementary Material

aakag008_Supplemental_material_v_1_2_3_1_26_2_finalpdf
