# Supplementary material for: Status on stroke and stroke care in Europe 2023: Stroke Service Tracker 2023 data based on 1,460,360 strokes in 47 European nations
Source: Eur Stroke J. 2026 Feb 19;11(2):aakag008. doi: 10.1093/esj/aakag008 (PMC12919429; doi:10.1093/esj/aakag008)
Supplement: aakag008_Supplemental_material_v_1_2_3_1_26_2_finalpdf [file aakag008_supplemental_material_v_1_2_3_1_26_2_finalpdf.pdf]

SUPPLEMENTAL TABLES

Table 1. Definition of key performance indicators (KPIs).

	Definition	Benchmark
KPI 1	A national stroke plan defining pathways, care and support after stroke, including pre-hospital phase, hospital stay, discharge and transition, and follow-up.	KPI 1met if confirmed and documented by link/upload of plan
KPI 2	At least one individual from the respective SSO (if existent) will be involved and supported, in an equal way, during the development of each country's national stroke plan or stroke-related guideline.	KPI 2 met if confirmed and documented by link to SSO and naming responsible person
KPI 3	A national strategy for multi-sectorial public health interventions that promote and facilitate a healthy lifestyle and risk factor control has been implemented.	KPI 3 met if confirmed and documented by link/upload of plan
KPI 4	Establishment of national- and regional-level systems for assessing and accrediting stroke clinical services, providing peer support for quality improvement, and making audit data available to the public**.**	KPI 4 met if confirmed and documented by link/upload of quality program
KPI 5	All stroke units and other stroke services independent of sector undergo quality auditing continuously or at regular intervals:<br>(a) Hospitals<br>(b) Other services.	KPI 5 met if confirmed to a and b
KPI 6	Access to stroke unit care for patients with acute stroke:<br>(a) Percentage admitted to stroke unit care	KPI 6 met if ≥ 90% of all stroke patients are admitted to stroke unit care based on high quality data.
KPI 7	Recanalisation treatment provided for patients with ischaemic stroke:<br>(a) Percentage of patients treated with IVT<br>(b) Percentage of patients treated with MT<br>(c) Median door-to needle times (IVT)<br>(d) Median door-to-groin times (MT).	KPI 7a met if IVT rate ≥ 20% based on high quality data.<br><br>KPI 7b met if MT-rate ≥ 7.5 % based on high quality data<br><br>KPI 7c met if DTN-time < 30 minutes based on high quality data<br><br>KPI 7d met if DTG-time < 60 minutes based on high quality data
KPI 8	Stroke units with mandatory access to: CT/MRI, vascular imaging, ECG, long-term ECG-monitoring,	KPI 8 is met if at least 6 /7 investigations are

	cardiac echo (TTE, TOE), dysphagia screening, and blood tests during stroke unit admission.	mandatory based on high quality data
KPI 9	Access to early stroke unit rehabilitation including early supported discharge.<br>(a) Mandatory access to early stroke unit rehabilitation<br>(b) mandatory access to early supported discharge.	KPI9a is met if early stroke unit rehabilitation is available in ≥ 90% of stroke units based on high quality data<br><br>KPI 9b is met if Early supported discharge is provided in ≥ 90% of stroke units based on high quality data
KPI 10	Access to basic secondary prevention, including antithrombotics, antihypertensives, statins and lifestyle advice.	KPI 10 is met if access is provided to all four based on high quality data
KPI 11	A binding, personalised, documented rehabilitation and sector transition plan is provided at the time of discharge.	KPI 11 is met if a plan is provided to at least 60% of patients based on high quality data
KPI 12	Follow-up at 3–6 months after the stroke incident, including a post-stroke checklist, functional assessment and referral for relevant interventions:<br>(a) Follow-up at 3–6 months<br>(b) Use of post stroke checklist, functional assessment and referral for relevant interventions at follow-up.	KPI 12a is met if at least 90% of patients are provided follow-up based on high quality data<br><br>KPI 12b is met if at least 90% of patients are followed up using a post-stroke check list
KPI 13	Percentage of patients in whom short-term survival (30 days) after stroke is monitored and at acceptable levels for:<br>(a) ischaemic stroke<br>(b) ICH<br>(c) all stroke.	KPI 13a is met if mortality within 30 days after ischemic stroke is <10% based on high quality data<br><br>KPI 13b is met if mortality within 30 days after haemorrhagic stroke is <30% based on high quality data

		KPI 13c is met if all stroke mortality within 30 days is <15%<br>Based on high quality data

CT: computed tomography; ECG: electrocardiography; ICH: intracerebral haemorrhage; IVT: intravenous thrombolysis; MRI: magnetic resonance imaging; MT: mechanical thrombectomy; SAH: subarachnoid haemorrhage; SSO: stroke support organisation; TOE: transoesophageal echocardiography; TTE: transthoracic echocardiography; DTN: door-to-needle time; DTG: door-to-groin time

SUPPLEMENTAL FIGURES

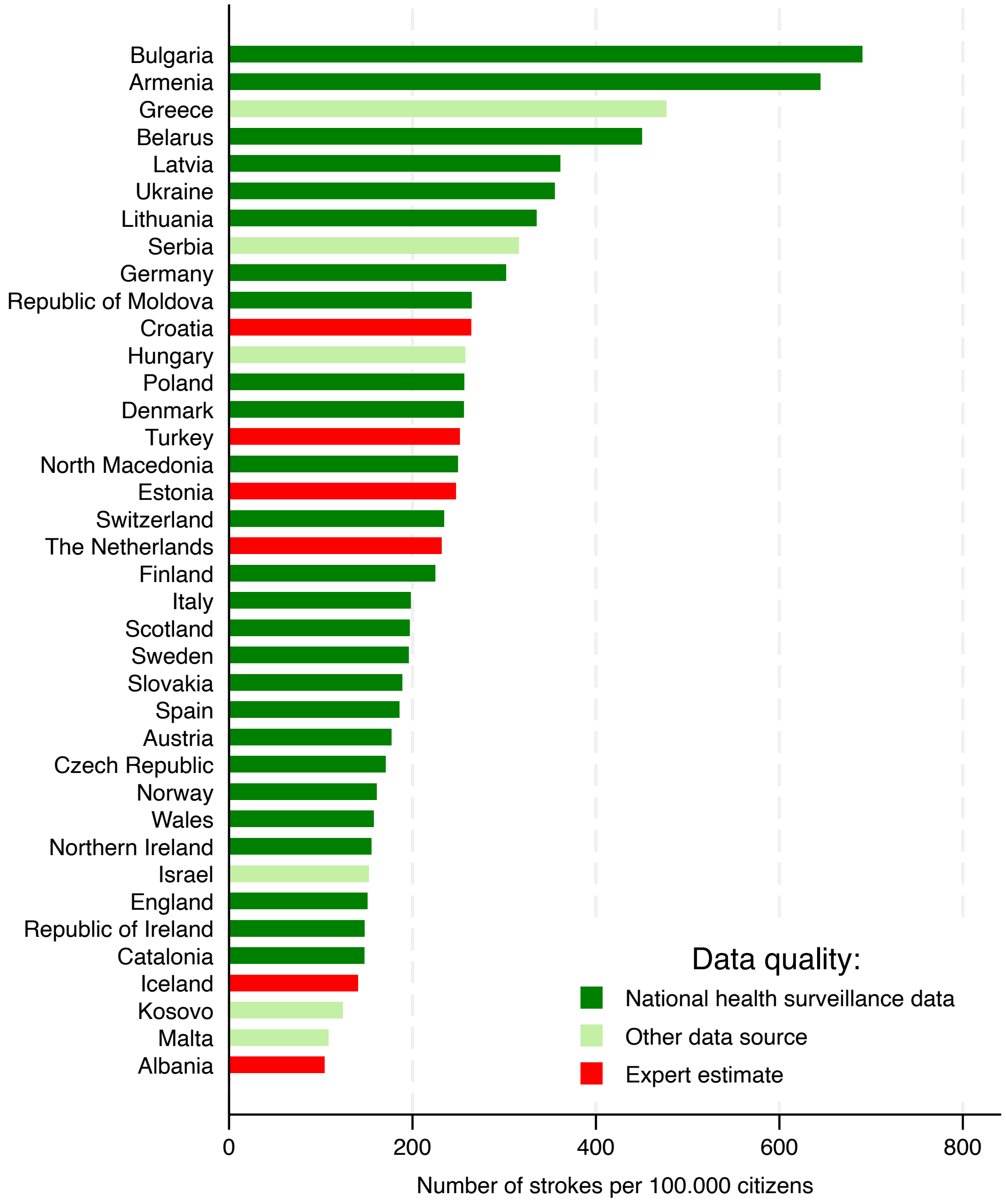

Supplementary figure 1. *Crude incidence of reported strokes*

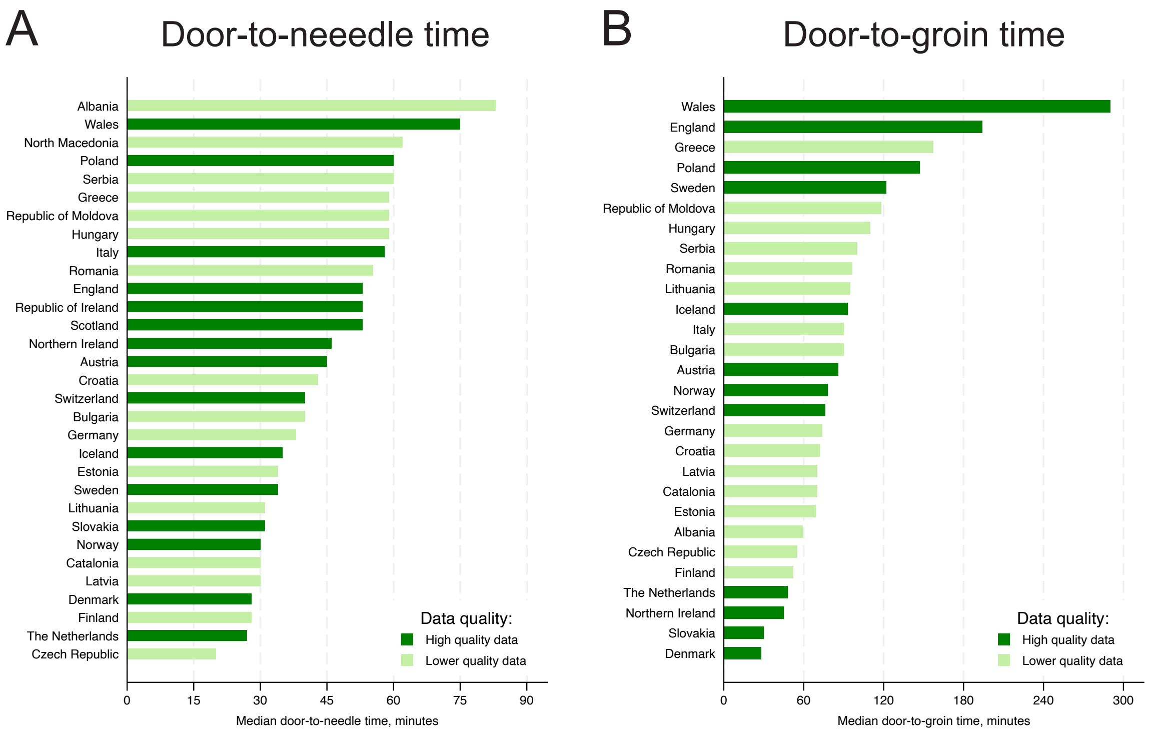

Supplementary figure 2. *National door-to-needle time (panel A) and door-to-groin time (panel B) in patients with ischaemic stroke receiving intravenous thrombolysis and mechanical thrombectomy, respectively.*

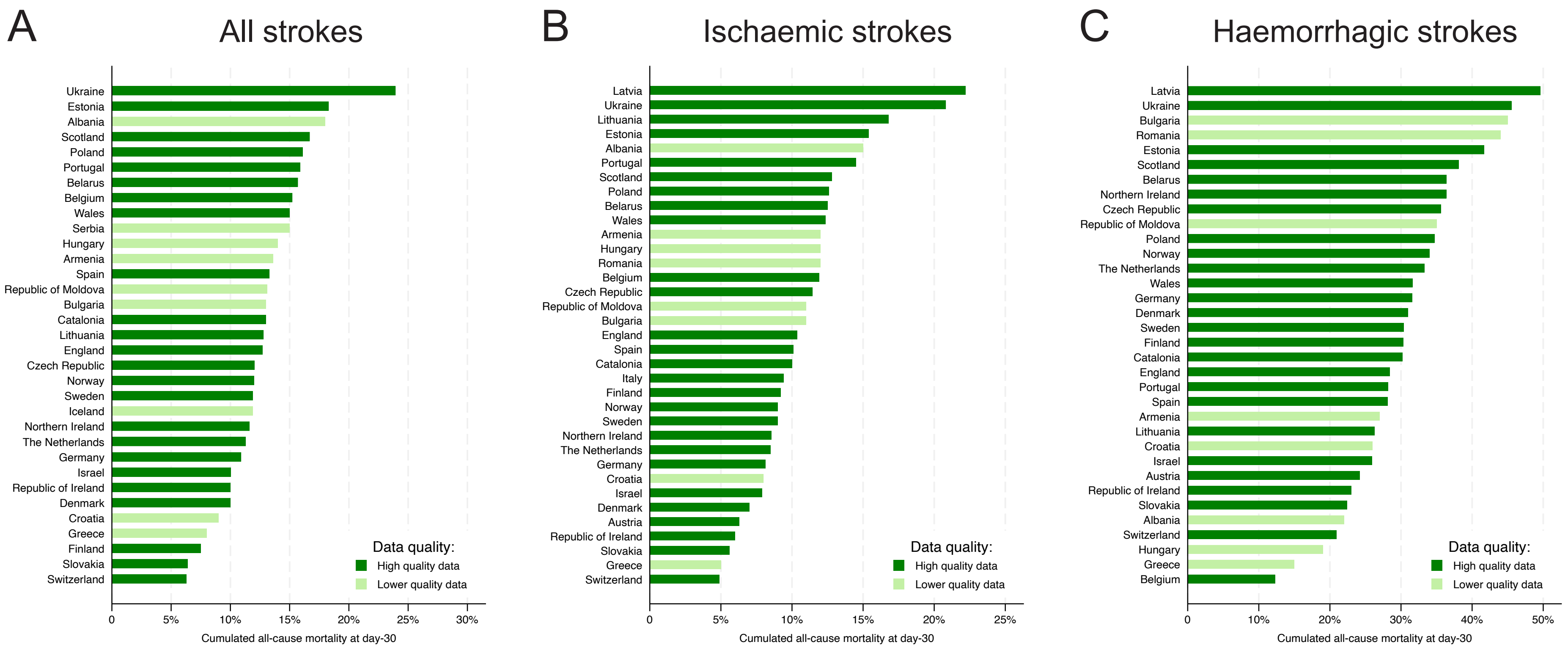

Supplementary figure 3. *Reported mortality within 30 days in all strokes (panel A), ischaemic stroke (panel B) or haemorrhagic stroke (panel C). I all cases discharge mortality was used if 30 days mortality was not available.*

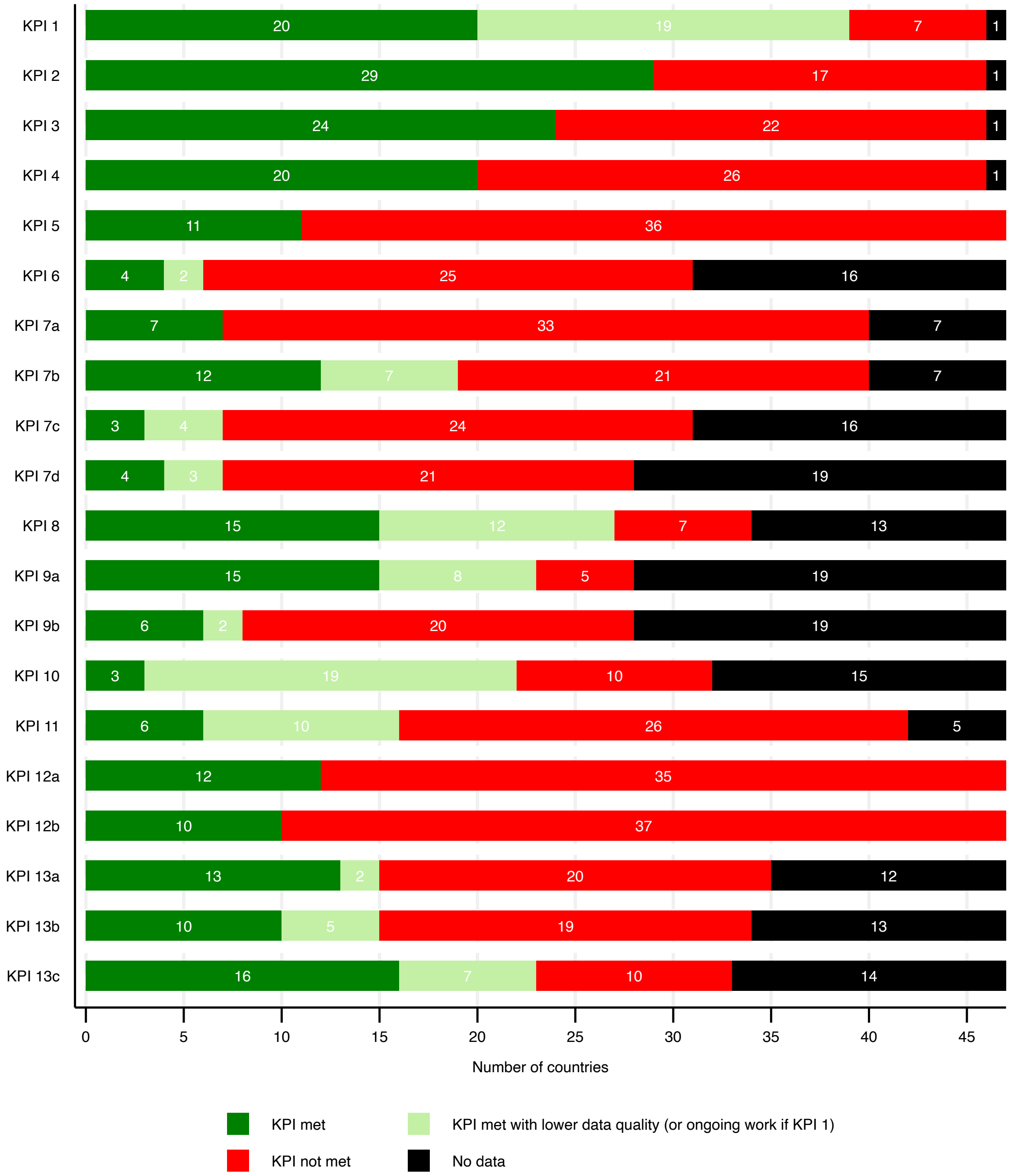

Supplementary figure 4. *Number of countries fulfilling the individual KPIs of SAP-E. In KPIs with subclassification, each response is counted as one.*
